# Phospholipid Metabolism in an Industry Microalga *Chlorella sorokiniana*: The Impact of Inoculum Sizes

**DOI:** 10.1371/journal.pone.0070827

**Published:** 2013-08-05

**Authors:** Shuhuan Lu, Jiangxin Wang, Qian Ma, Jie Yang, Xia Li, Ying-Jin Yuan

**Affiliations:** 1 Key Laboratory of Systems Bioengineering, Ministry of Education and Department of Pharmaceutical Engineering, School of Chemical Engineering and Technology, Tianjin University, Tianjin, P. R. China; 2 CABIO Bioengineering (Wuhan) Co., Ltd, Wuhan, Hubei, P.R. China; National Institute for Viral Disease Control and Prevention, CDC, China, China

## Abstract

*Chlorella sorokiniana* is an important industry microalga potential for biofuel production. Inoculum size is one of the important factors in algal large-scale culture, and has great effects on the growth, lipid accumulation and metabolism of microalgae. As the first barrier of cell contents, membrane plays a vital role in algal inoculum-related metabolism. The knowledge of phospholipids, the main membrane component and high accumulation of phospholipids as the major content of total lipids mass in some microalgae, is necessary to understand the role of membrane in cell growth and metabolism under different inoculum density. Profiling of *C. sorokiniana* phospholipids with LC-MS led to the identification of 119 phospholipid species. To discover the phospholipid molecules most related to change of inoculum sizes, Partial Least Squares Discriminant Analysis (PLS-DA) was employed and the results revealed that inoculum sizes significantly affected phospholipid profiling. Phosphatidylglycerol (PG), phosphatidyl- ethanolamine (PE) and several phosphatidylcholine (PC) species might play an important role under our experimental conditions. Further analysis of these biomarkers indicated that cell membrane status of *C. sorokiniana* might play an important role in the adaption to the inoculum sizes. And the culture with inoculum size of 1×10^6^ cells mL^−1^ presented the best membrane status with the highest content of PC and PG, and the lowest content of PE. We discovered that the inoculum size of 1×10^6^ cells mL^−1^ might provide the best growth condition for *C. sorokiniana*. Also we proposed that PG, PE and several PC may play an important role in inoculum-related metabolism in *C. sorokiniana*, which may work through thylakoid membrane and photosynthetic pathway. Thus this study would provide more potential targets for metabolic engineering to improve biofuel production and productivity in microalgae.

## Introduction

Currently, the demand of energy has risen rapidly because of the modernization and the industrialization of the world. However, the fossil fuels were considered as unsustainable and causing environmental pollution [1]. The renewable energies have become research hotspot in recent years. Amongst the renewable energies,, biofuels derived from microorganism is one of the most promising alternative to fossil fuel-based energy sources [2], [3]. Researchers studied microalgae cultivation and microalgal biofuel production from different directions, such as expanding the new application of algae, system screening of valuable algae strains [4], optimization of process conditions [5], [6], investigation of metabolic pathways [7], [8], metabolic engineering [9], [10], genome analysis [11] and so on. The green phototrophic microalga *Chlorella sorokiniana* is a potential algal candidate for biofuel production, which casts most industry interests due to its fast growth rate, easy cultivation, and wide adaptability [12], [13], [14]. It is well known that oil-rich microalgae species are the most productive biofuel crops which could provide 10∼100 times higher biomass and oil yield than land oil crops [15], [16]. Furthermore, several scientists reported positive results on the large culture of algae for biofuels [17], [18]. The algae oils are mainly composed of triacylglycerols (TAG), diacylglycerols (DAG), free fatty acids (FFA) and phospholipids (PL), and TAG contributes more than 80% of algae lipid mass. Oils from algae are generally transesterificationto yield fatty acid methyl esters (FAME) [19], [20]. Furthermore, Previous studies have shown that phospholipid synthesis precursors, DAG and FFA, could not only be derived from TAG, but also from membrane phospholipids [21].

However, microalgal biofuels could not make an impact on the fuel market yet due to high producing cost [22], [23]. One of the vital cost factor is low cell density culture resulting in long cycle time and high cost of the dewatering process in downstream [24]. Besides, low cell density culture also hamper the systematic screening for new compounds from pure cultures [24] and axenic culture of algae [25]. Although high cell density culture could solve certain problems mentioned above, it also has inevitable negative effects on cell culture with cell stress and typically light and nutrient limitation [26]. Therefore, an optimized process is still highly needed to improve the cell density in the culture to achieve the highest biofuel productivity. Changing initial cell density has been reported as an efficient solution to control cell density of culture [27], [28], and inoculum-associated growth and target product improvements also have been reported [20], [29]. However, extensive investigation into the nature of lipid accumulation are still limited, and metabolic mechanisms of lipid biosynthesis upon inoculum sizes remains poorly understood [20], [30]. Previously, we demonstrated that inoculum sizes (1×10^4^–1×10^7^ cells mL^−1^) significantly affected cell growth, lipid accumulation, protein and metabolism regulation in a photoautotrophic culture of *Chlorella sorokiniana*, and lipid production was tightly related to photosynthetical carbon fixation metabolism [28], [31]. Nevertheless, cellular response to inoculum sizes has not been fully explored yet.

Numerous factors affect photosynthetic microorganism’s ability to adapt high cell concentrations, such as pH, light condition, cell contact frequency and so on, which all must across the essential barrier between cell interior and environment, membrane, to affect the cell. Accordingly, the plasma membrane appears to be a primary target and communication platform of perturbation effects of inoculum sizes on cells. Structure and composition of phospholipids strongly influence the physicochemical properties and dynamical properties of membranes [32], [33], [34]. Furthermore, phospholipids are now known to play a vital role in algal cellular signaling and cell-cell interactions [35]. A large body of evidence established that phospholipids on algal membranes are fundamental to lots of important biological processes, such as stress response, photosynthesis, and so on [36], [37]. For instance, there is strong evidence that phospholipid distribution of yeast, in part, contributes to its tolerance to increasing cell density [27]. Thus knowledge of phospholipid changes associated with the biological characteristics of membrane is essential to understand the potential mechanisms of how *C. sorokiniana* utilizes these molecules to adapt to inoculum size changes. Thus, dissecting the details of phospholipid changes would provide a clearer understanding of the inoculum sizes-dependent metabolism in this industry microalga.

To elucidate involvement of phospholipids in the cellular responses to inoculum size changes in *C. sorokiniana*, LC-MS based approach was employed for phospholipid profiling, followed by Partial Least Squares Discriminant Analysis (PLS-DA) for data classification and potential biomarkers selection. In this study, three types of phospholipid molecules were identified as biomarkers, including phosphatidylglycerol (PG), phosphatidylethanolamine (PE) and phosphatidylcholine (PC). Their functions in relation to regulation of cell membrane stability, signal transduction and photosynthesis efficiency under different inoculum sizes were also discussed**.**

## Methods

### Reagents and Materials

Phospholipid internal standards in this paper were as follows: 1,2-dilauroyl-*sn*-glycero-3-phospho(1′-*rac*-glycerol)(sodium salt)(PG12∶0/12∶0), 1,2-dilauroyl-*sn*-glycero-3-phosphoethanolamine(PE12∶0/12∶0), 1,2-dilauroyl-*sn*-glycero-3-phosphocholine(PC12∶0/12∶0), 1,2-dipalmitoyl-*sn*-glycero-3-phospho-(1′-*myo*-inositol)(ammonium salt)(PI16∶0/16∶0), 1,2-dilauroyl-*sn*-glycero-3-phospho-L-serine (sodium salt)(PS12∶0/12∶0), 1,2-dimyristoyl-*sn*-glycero-3-phosphate (sodium salt) (PA14∶0/14∶0). All these internal standards were purchased from Avanti Polar Lipids (Alabaster, AL. USA) and prepared in chloroform-methanol(1∶1, v/v) to a final concentration of 1µg mL^−1^. High-performance liquid chromatography(HPLC)-grade chloroform and methanol were procured from Merck(Darmstadt, Germany)and Ammonium hydroxide (28%) was obtained from J&K Chemical (Beijing, China). Except for those were noted, reagents and solvents were purchased from Sigma-Aldrich (St. Louis, MO) at the highest grade commercially available, and all water used was obtained from a Milli-Q Synthesis (Millipore, Billerica, MA).

### Algal Strain and Culture Conditions

The *C. sorokiniana* strain was obtained from Dr. Dingji Shi (Tianjin University of Science Technology, Tianjin, China). Algae cultivation was carried out as previously described. Briefly, cells were inoculated into 250 mL flask containing 150 mL BG11 medium with shaking (135 rpm) under continuous illumination provided by cool-white fluorescent lights (65 µmol m^−2 ^s^−1^) at 25°C. Growth was determined by counting cells with blood cell counting plate under light microscopy. Four inoculum sizes used in initial cell density assay were 1×10^4^, 1×10^5^, 1×10^6^ and 1×10^7^ cells mL^−1^ and cells were harvested at two phases, the exponential and stationary phase, respectively. Notably, the samples here were divided into 8 classes (see below for details) and named after different inoculum sizes and harvest growth phases. For example, cells with inoculum sizes of 1×10^4^ cells mL^−1^ were named as IN104, and cells at exponential phase and stationary phase were specified as “E” and “S”, respectively. The cells were collected and frozen at −70°C in a Labconco freeze dryer overnight and kept in labeled tubes at −80°C till further investigation.

### Phospholipids Extraction from *C. sorokiniana*

Phospholipid extraction was performed as described by Bligh [38] and Chen [39] with slight modifications. Briefly, 20 mg lyophilized cells were suspended in 0.75 mL chloroform and 0.3 mL ultrapure water and shaken (100 rpm) for 1 h at room temperature (RT). Subsequently, 2 mL of lipid extract buffer (LEB, chloroform-methanol, 2∶1, v/v, with 0.1% (w/v) butylated hydroxytoluene) was added, followed with another shaking at RT for 30 min. The chloroform layer was collected, and another three extractions were repeated by adding 2 mL LEB into the aqueous layer and shaking for 30 min. All chloroform layers from four times of extraction were combined into one tube and washed with 0.5 mL 1 M KCl and 1 mL ultrapure water sequentially. The solvents were then removed by evaporating under vacuum with rotary evaporator (BUCH Vacuum Rotavapor, Germany) at 35°C and the final pellets were stored at −40°C for further analysis.

### LC-MS Analysis

The LC-MS analysis of phospholipids was carried out on a Waters Alliance 2695e HPLC/autosampler system (Milford, MA, USA) coupled to a Waters Quattro micro API triple quadrupole MS system (Micromass, Manchester, UK) [40], [41]. The total phospholipid was separated on a Venusil XBP Silica column (150 mm×2.1 mm i.d., 5 µm, Agela, DE, USA) with a column temperature of 25°C. The mobile phase A was chloroform/methanol/28% ammonium hydroxide (89.5/10/0.5, v/v), and mobile phase B was chloroform/methanol/water/28% ammonium hydroxide (55/39/5.5/0.5, v/v). The linear gradient program was set as follows: 0–7 min, 10–23% B; 7–10 min, 23% B; 10–15 min, 23–28% B; 15–20 min, 28–34% B; 20–25 min, 34–40% B; 25–45 min, 40–50% B; 45–50 min, 50% B; 50–60 min, 50–10% B; 60–70 min, 10% B. The injection volume was 5 µl, and the flow rate was 0.2 ml min^−1^.

Semi-quantification of identified phospholipids species was carried out on the tandem quadrupole mass spectrometer under negative electrospray ionization (ESI) condition by full scan within a single acquisition. For full scan, the mass ranged from m/z 550 to 1000 with a scan time of 0.45 s and inter-scan delay of 0.02 s; the voltage of capillary, extractor and RF (radio frequency) lens were 3 kV, 3 V, and 0.1 V, respectively; the source and desolvation temperature were 100°C and 350°C respectively; the flow rates of nitrogen as desolvation gas and cone gas were 400 L h^−1^ and 50 L h^−1^, respectively. For semi-quantification of individual phospholipids molecules, extracted ion chromatograms (EIC) was applied to integrate peak areas by using ApexTrackTM peak detection algorithm in Masslynx (Version4.1) Quanlynx Applications Manager after correction for the contribution of the 13C isotope effect. Each ion peak area was normalized to that of the corresponding internal standard and the dry weight of cell (DWC), expressed by nmol phospholipids (mg DWC)^−1^.

### Multivariate Statistical Analysis

In this work, one pattern recognition method PLS-DA was performed to investigate correlations between different samples and identify potential key phospholipids involved in inoculum-associated response. The data in the multivariate statistical analysis was set as X-matrix consisted of 119 individual phospholipid species from 53 samples. The data was handled by centre and pareto scaling prior to PLS-DA analysis with SIMCA-P Demo (Umetrics AB, Sweden). The quality of PLS model was evaluated by three parameters: R2(X), R2(Y) and Q2(Y), resulting in a valid and clear phospholipid profile separation between the algal samples analyzed. This statistical evaluation allows differentiate and characterize the sample and gives a possible biological interpretation of data. The range of the three parameters is 0–1, and the higher the value of these parameters, the better the explanation and reliable predictive ability of the model.

### Statistics Analysis

Experimental data in this paper were obtained from at least six replicates for each treatment, and values were shown as mean ± standard deviation. Analysis of variance (ANOVA) was applied to ascertain the significant of differences, and the p-values less than 0.05 were considered as significant (*) and the p-values less than 0.005 were considered as highly significant (**).

## Results and Discussion

### Identification and Quantification of Phospholipid (PL) Species

**Figure 1:**
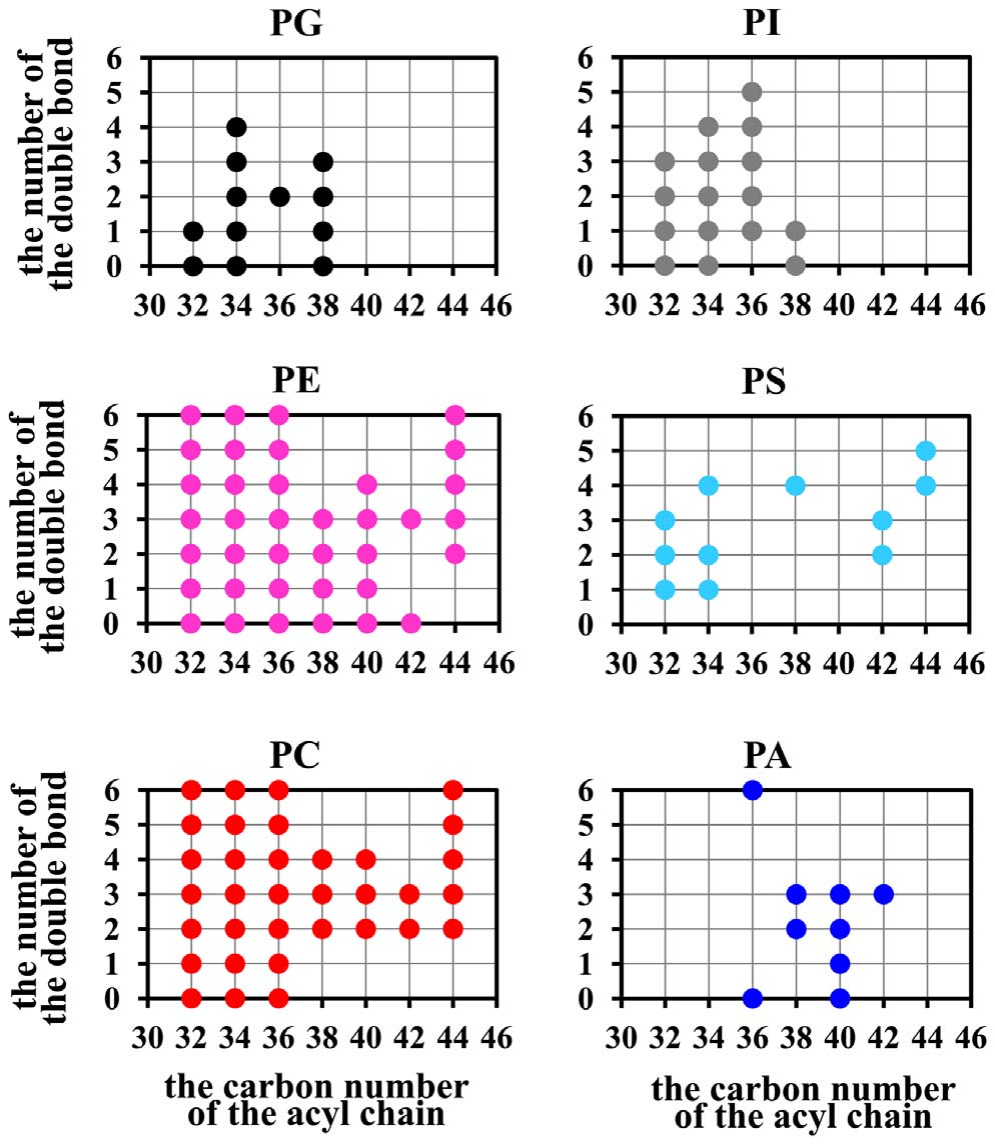
The molecular species profile of phosphlipids from *C.* *sorokiniana*.

**Figure 2:**
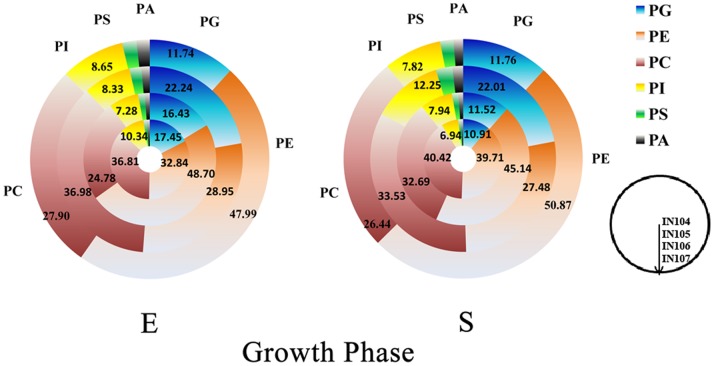
The phosphlipid distribution of *C.* *sorokiniana* under different inoculum sizes.

All phospholipid samples extracted from *C. sorokiniana* were analyzed under the optimal LC-MS conditions. The order of elution was PG, PE, PC, phosphatidylinositol(PI), phosphatidylserine(PS) and phosphatidic acid(PA), as six main phospholipid classes in *C. sorokiniana* (Figure 1). In this study, total 119 phospholipid species comprised of 12 PG, 37 PC, 16 PI, 11 PS and 9 PA were detected. The number of carbon atoms of two fatty acyl chains of PLs of *C. sorokiniana* ranged from 32 to 44 while the number of carbon-carbon double bonds of acyl chain ranged from 0 to 6. The result was in line with distribution of length and degree of unsaturation fatty acids in *C. sorokiniana* [28]. Nevertheless, composition of acyl chains in treatments was obviously different compared to each other (Figure 2). The diversity of acyl chain moieties of PE and PC were much more extensive than the others. Both PI and PG contained short chain fatty acids (C32–C38), much shorter than those of PA (C36–C42). Researchers also identified PG, PE, PC and PI from another green alga *Dunaliella salina*, and pointed out that main acyl chains were C14∶0, C16∶0, C16∶1, C18∶0, C18∶1, C18∶2 and C18∶3 [42]. Another report also indicated that the main phospholipids species in a diatom *Nitzschia laevis* were PC, lysophosphatidylcholine(LPC), PI, PG and DPG with acyl chain ranged from 14∶0 to C20∶5, with dominant C16 acyl chains [43].

As shown in Figure 2, the two concentric circles displayed the quantitative analysis of *C. sorokiniana* samples and presented phospholipid class distribution for each inoculum culture at exponential and stationary phase, respectively. These results allowed observation of variation in PL classes of *C. sorokiniana* extracts. It’s clear that PC, PE and PG constituted major percentage (83.02–91.04%) of total phospholipids in this microalga. Although inoculum sizes had no effect on the composition of phospholipids (119 PL detected in all samples), it changed the content of each phospholipid class significantly (Figure 1). Compared with other *C. sorokiniana* culture conditions, IN106 showed a different profile with a sharp increase of PG, a dramatic decrease of PE and a minor increase of PC. In details, content of PG under IN106 at the exponential and stationary phase was 22.24% and 22.01%, respectively. Interestingly, PG amounts detected in other cultures showed a reduction of 21.52–47.18% and 46.58–50.42% compared to IN106-E and IN106-S, respectively. These results evidently illustrated the phospholipid compositional differences between IN106 and the others, while similarities between IN104 and IN105 suggested similar phospholipid metabolism trend when compared with IN106-E and IN106-S, respectively. A similar trend was observed in IN107, which was significantly lower than the others. It can be suggested that IN106 had distinguished phospholipid metabolism different from the other treatments.

Our results were consistent with several recent reports where major phospholipids in microalgae species were PC, together with PE and PG, and less amounts of PS, PI, PA and diphosphatidylglycerol (DPG) [44], [45]. Nonetheless, significant difference of phospholipids compositions of microalge from species to species was also noticed. For instance, some recent reports indicated that PC, PE, PG and PI were predominant in *Ahnfeltia tobuchiensis*, PC, PE, PA, PG and PI in *Laminaria japonica*, PE, PG and PI in *Sargassum pallidum*, and PS, PE, PA, PG and PI in *Ulva fenestrata* [44], PC, LPC, PG, PE, LPE, PI and LPI in *Nannochloropsis oculata* [46], and PC with PIF, PG, DPG and LPC in *Nitzschia laevis* [47]. The phospholipid compositions determine the membrane physiological characteristics of mircoalgae, as variations in phospholipid headgroup could cause change of membrane biophysical properties, such as membrane intrinsic curvature, protein distribution and so on [48]. Our current phospholipidomic study indicated that PE and PG were the key phospholipid molecules causing phospholipid profile alternations under different inoculum sizes (Figure 2), suggesting the importance of membrane density and fluidity of photosynthesis-related thylakoid membrane in metabolism of microalgae under different inoculum sizes.

### Effects of Inoculum Sizes on Acyl Chain Length and Unsaturation of PL Species in *C. sorokiniana*

**Figure 3:**
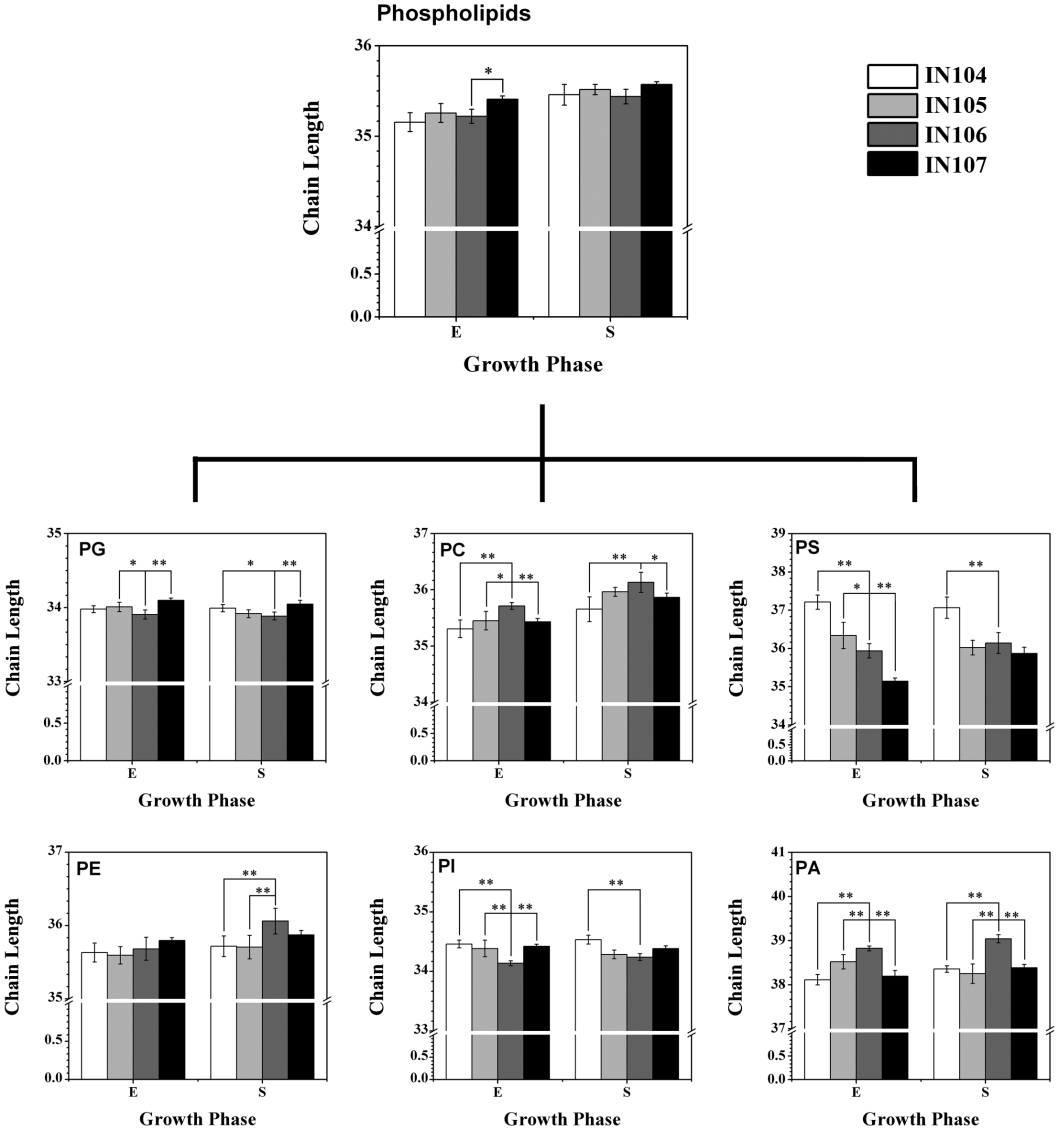
The chain lenth of phospholipids in *C.* *sorokiniana* under different inoculum sizes.

Besides the phospholipid headgroup, acyl chains also characterize membrane structure and dynamics, and thus membrane-associated biological processes [49], [50]. Our results showed that inoculum sizes had little impact on the average length of the acyl chains of total phospholipids with a range from 35.15 to 35.57. However, a close look at average acyl chain length (CL) of different phospholipid classes showed significant difference. For instance, PG and PI had the shortest CL, followed by PE, PC and PS. PA showed a longer CL (112.00–115.21%) compared with that of PG. Furthermore, the inoculum-associated CL varied significantly according to the phospholipids classes (Figure 3). For example, compared with the other culture conditions, IN106 had the shortest CL of PG, PI, and the longest CL of PC and PA. The CL level of PE was relatively stable in the exponential phase, with the maximum length (36.06±0.17) in IN106-S. Interestingly, the CL of PS was decreased with increasing inoculum sizes, suggesting a possible different trend compared with other phospholipid classes.

The influence of CL on biophysical properties of membranes, such as stability and fluidity, has been studied for years [50], [51], [52]. Zhao and Feng have established an artificial membrane system by PC with various hydrophobic chain moieties, and found that the net van der Waals interaction between acyl chains increased with increasing CL, leading to less stability and decreased bilayer intermolecular spacing [52]. Niemelä and coworkers also investigated the influence of CL on bilayers, and revealed that increasing CL would increase bilayer thickness and further interdigitation across the bilayer center [51]. High concentration of long aycl chain fatty acid residues of membrane lipids could result in a high rigidity, which is mainly present as a crystalline, “solid” phase [53]. In IN106, the variation of CL of PG and PI could cause an increase of the membrane fluidity, while that of PC, PE and PA showed an opposite tendency. This might be arisen partly from dissimilar metabolic pathway for different phospholipid classes. In microalgae, PI and PG can only be synthesized from cytidyldiphosphate-diacylglycerol (CDP-DAG) pathway from PA, while both CDP-DAG and Kennedy pathways are involved in production of PC and PE (http://www.kegg.jp), which might explain why these phospholipids classes had different CL variation under different inoculum size cultures of *C. sorokiniana*. Another reason might be different biological characters of those phospholipids. For instance, PG is the only phospholipid presents in significant quantities in thylakoid membranes while other phospholipids locate in extra-chloroplast membranes. A large body of evidence demonstrated that PG played an important role in microalgal photosynthesis [54], thus increasing the fluidity of thylakoid membrane might be helpful for the improvement of electron transport rate and photosynthetic efficiency [55].

**Figure 4:**
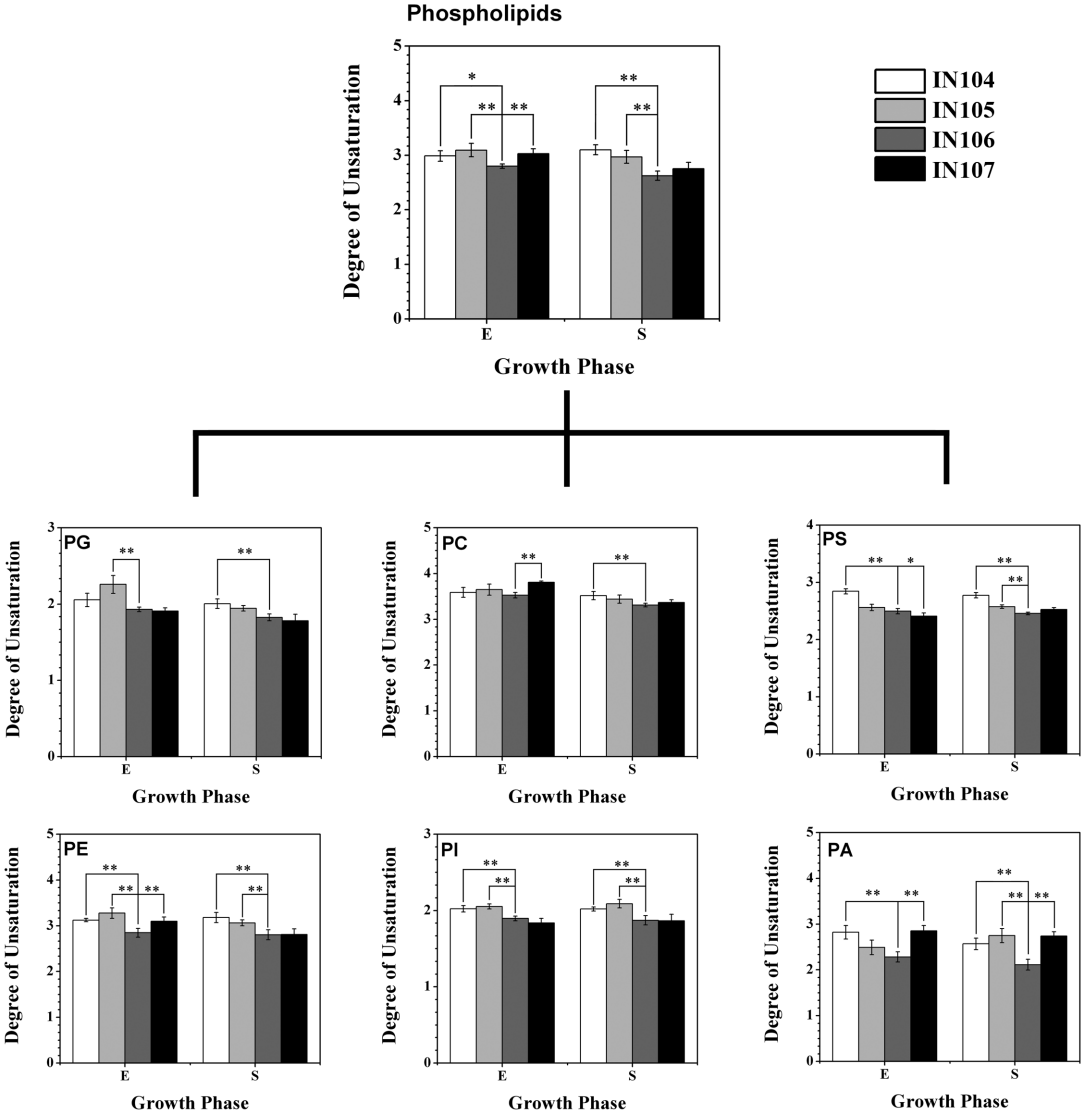
The degree of unsaturation of phospholipid in *C.* *sorokiniana* under different inoculum sizes.

Interestingly, in this study levels of acyl chain unsaturation of phospholipids were in correlation with inoculum sizes, and the lowest degree of unsaturation (DU) of all detected phospholipids was observed in IN106, resulting in much lower total DU of phospholipids in IN106 than those from other cultures (Figure 4). High DU of acyl chain could increase spatial configuration of phospholipid molecules and membrane fluidity which helps microorganisms in adaptation to low environmental temperature [56]. A recent report showed that exposure to saturated fatty acids at concentrations leading to endoplasmic reticulum membrane phospholipid remodeling would inhibit oxysterol activity [57]. Lin also found DU of acyl chains in bilayer could affect the sterol partitioning between lipid domains in plasma membrane [58]. Additionally, the variation of DU of specific phospholipids was reported in lots of studies. Take PG as an example, Sakamoto has reported that DU of PG acyl chains in chloroplasts strongly correlated with the chilling sensitivity of tobacco plants [59]. Ivanov also discussed the physiological role of the decreasing of DU of PG in down-regulation of photosystem I (PSI), modulation of the capacity of PSI-dependent cyclic electron flows, and distribution of excitation light energy in tobacco plants under photoinhibitory conditions under low temperatures [60]. These previous studies revealed that DU of phospholipids closely related to the biological characteristics of the membrane. Therefore, the decrease of DU of phospholipids in IN106 in the present work suggests a possible membrane adaption to the inoculum sizes.

### Identification of Potential Phospholipid Biomarker

**Figure 5:**
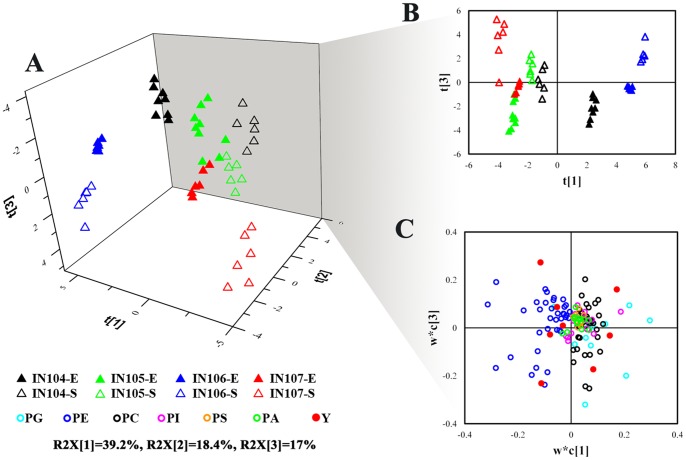
PLS-DA model based on the entire phospholipid profile in *C.* *sorokiniana*. (a) PLS-DA 3D score plot distinguishing *C. sorokiniana* grown under different inoculum sizes (R2X [1] = 39.2%; R2X [2] = 18.4%; R2X [3] = 17%). (b) PLS-DA score plot t [1]–t [3] indicating the separation between different groups; (c) PLS-DA loading plot w*c [1]−w*c [2] explaining the separation above.

To determine whether phospholipid profiling analysis could distinguish the inoculum size cultures, PLS-DA model was carried out to investigate inoculum-dependent phospholipid characteristics of *C. sorokiniana*. As shown in Figure 5, PLS-DA was performed to discriminate different inoculum sizes in 53 samples with available phospholipids information. The sample classification was determined by inoculum sizes and growth phases. The values of the first three principal components showed a small variation within the same class and samples in the same growth phase were grouped together on the three-dimensional PLS-DA score plot, indicating a high reproducibility of LC-MS analysis of phospholipid profile in this study (Figure 5a). Additionally, the PLS-DA score plot for the first and third components was examined in details (Figure 5b). The remarkable separation among samples collected at different growth conditions both in t1 and t3, reflected a high reproduction within the same class. IN106-E, IN106-S and IN104-E dominated and positively correlated to the first component (t1), whereas the third component separated samples based on the harvesting condition. Nevertheless, overlaps were still observed among different classes. IN this study, the PLS-DA model successfully categorized samples and the loading plot provided an insight into the changes of phospholipid among different classes.The loading plot of PLS-DA also illustrated the potential phospholipid biomarkers which definitively contribute to the classifications. Consecutively, the membrane metabolic difference observed all along the growth time and culture can be distinguished by the phospholipid profile, especially the samples grown under IN106. Considering the similar result obtained from a parallel proteomic analysis [31], we proposed that the culture with the inoculum sizes of 1×10^6^ cells mL^−1^ had a distinguished difference compared to the other inoculum sizes subjected to its unique phospholipid composition.

In an effort to get an insight into the most vital phospholipid in response of *C. sorokiniana* to incoulum size, we conducted the PLS-DA analysis to reveal potential biomarkers (Figure 5c). The PLS-DA loading plot w*c [1]–w*c [3] revealed the contribution of each phospholipid species to the model and enriched the ones most correlated to the first and third components as the potential biomarkers. And then a deeper investigation was applied to the most potentially relevant biomarkers.

**Figure 6:**
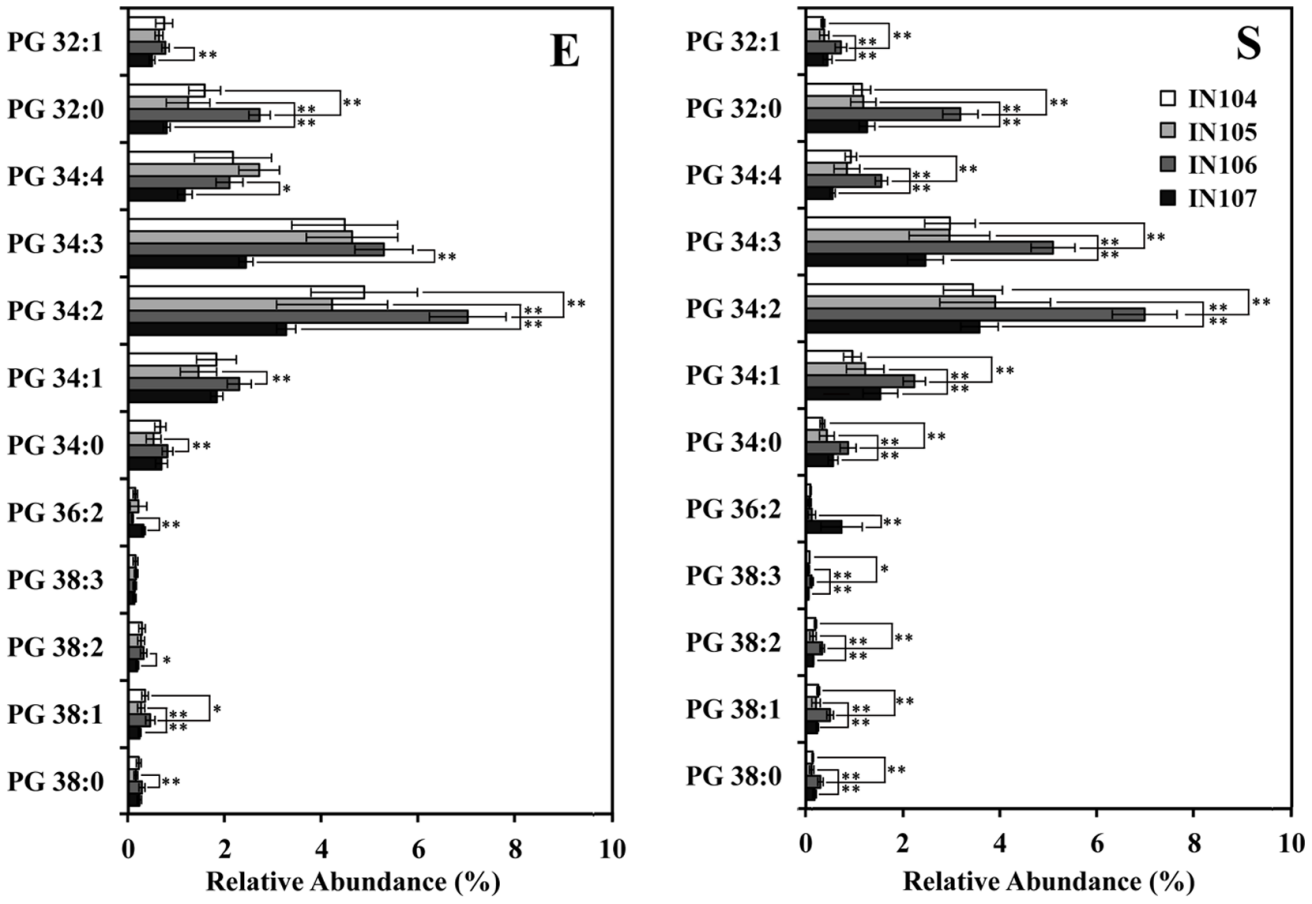
The biomarker PG content of *C.* *sorokiniana* under different inoculum sizes. Error bars represent standard error (n≥6). **, *p*<0.005.

**Figure 7:**
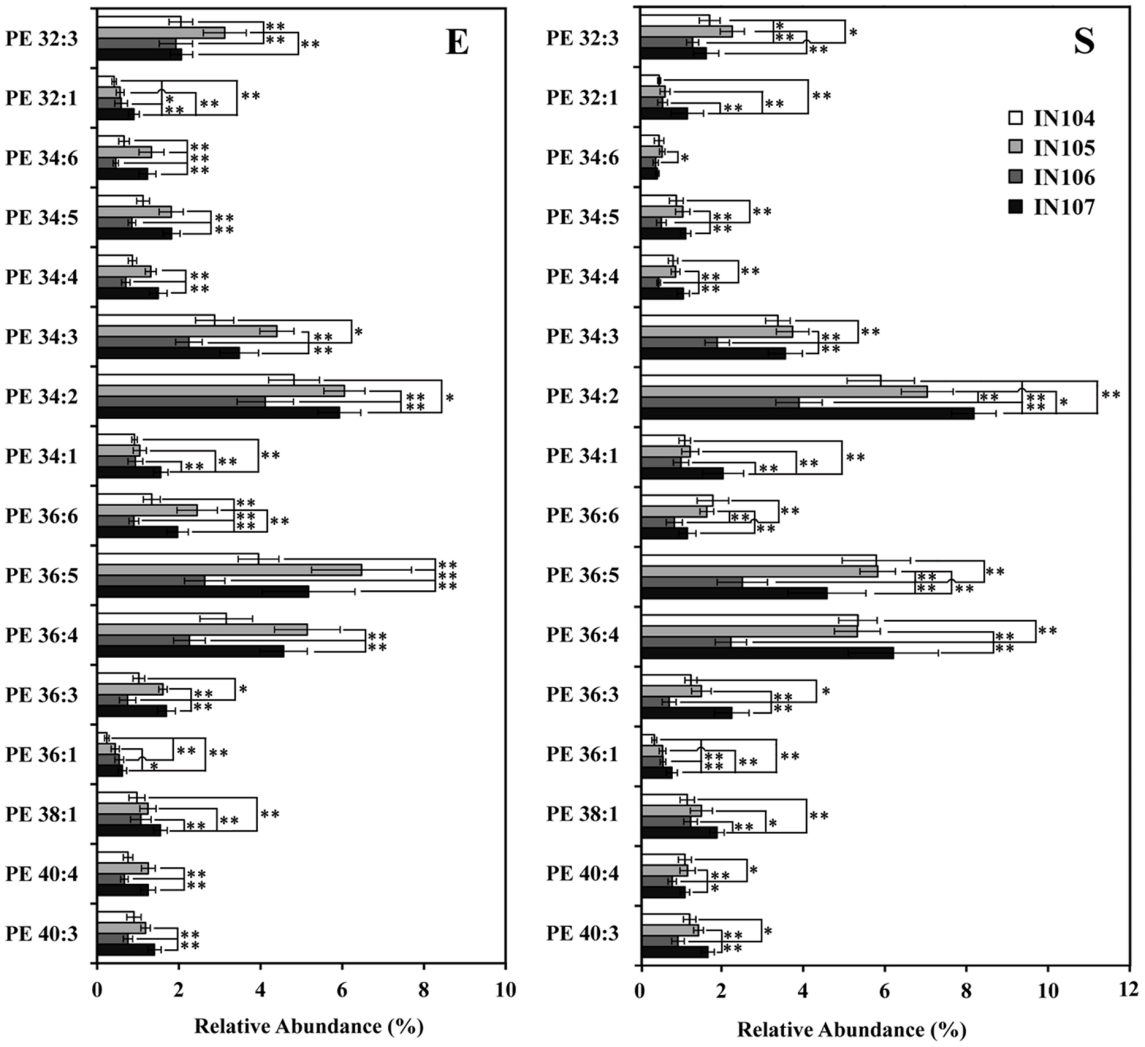
The biomarker PE content of *C.* *sorokiniana* under different inoculum sizes. Error bars represent standard error (n≥6). **, *p*<0.005.

**Figure 8:**
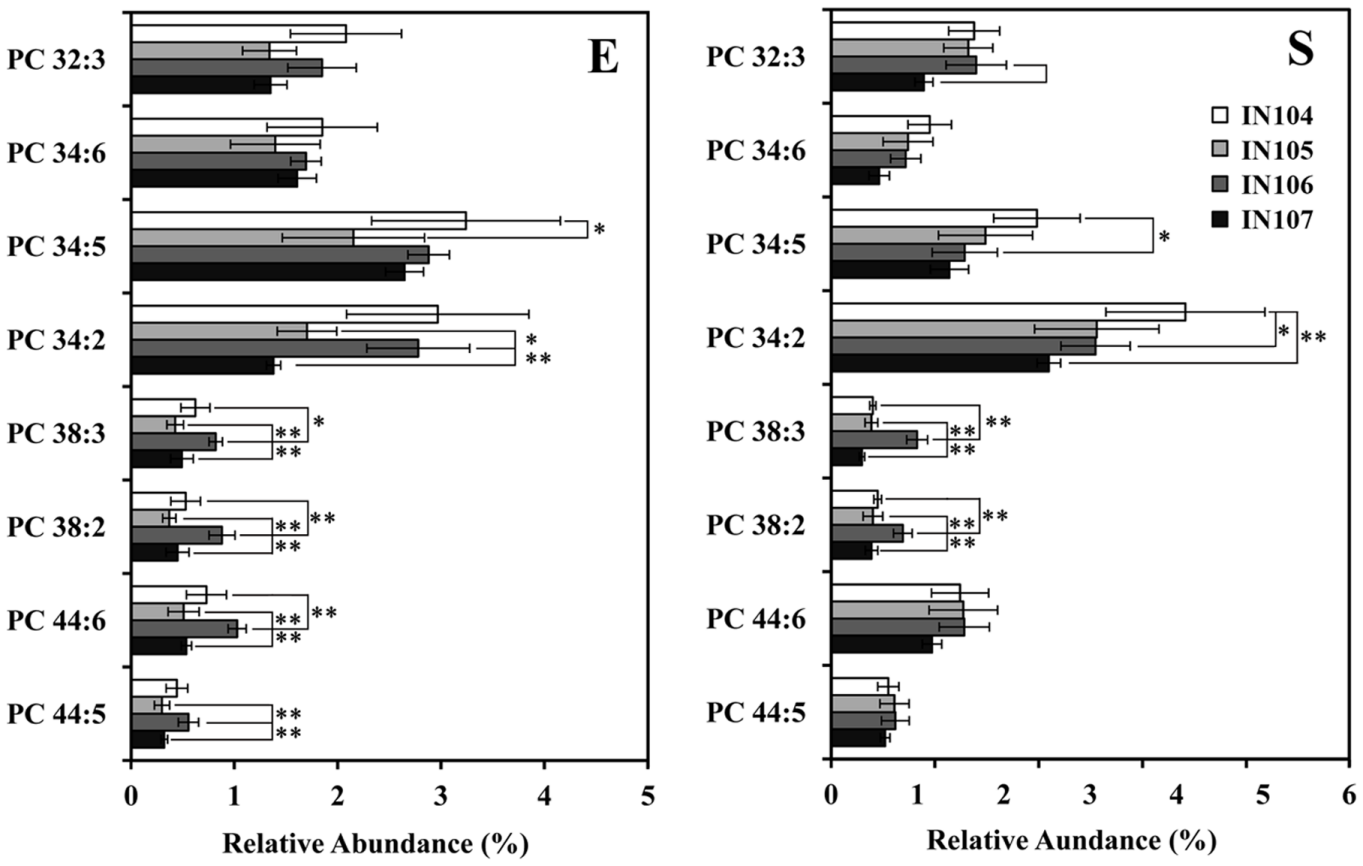
The biomarker PC content of *C.* *sorokiniana* under different inoculum sizes. Error bars represent standard error (n≥6). **, *p*<0.005.

Obviously, the content differences of all phospholipid biomarkers identified in this PLS-DA model were caused by responding to the initial cell density, which related to the cell membrane function of adaptation to different inoculums sizes. As shown in Figures 6, 7, 8, the biomarkers in this system have been shown to be PG, PE and PC. Variations in the content of these molecules yielded different membrane status that cells utilize to adapt to their initial cell densities. The results verified that *C. sorokiniana* had developed mechanisms of altering quantities and composition of phospholipids, especially those of PG, PE and PC, to cope with different inoculum sizes.

### Effect of Inoculum Sizes on Content of Potential Phospholipid Biomarkers

According to the PLS-DA analysis, the inoculum-dependent differential abundances among major PLs were PG, PE and PC. PG was the third most abundant class of phospholipids in *C. sorokiniana* (Figure 2). The highest concentrations of PG species observed in *C. sorokiniana* were PG34∶2 and PG34∶3. Furthermore, almost all PG molecules reached peak values in IN106 (Figure 6). These also explained the change of total concentration of lipids in *C. sorokiniana* culture [28].

Figure 7 displayed the change of vital PE molecules under different inoculum sizes. The PE species were another group of key phospholipids in inoculum-dependent culture, and also consist of the most diverse phospholipid class in terms of types of acyl chains and the second most abundant phospholipid class observed in *C. sorokiniana* (Figure 1). As shown in Figure 8, PE34∶2 was the most abundant PE species among all cultures and significantly reduced in IN106 when compared with others. Similarly, significant decrease of other PE species was also observed in IN106 (*p*<0.005). Additionally, PE32∶1, PE34∶2, PE34∶1, PE36∶1 and PE38∶1 were positively related to the third component in Figure 4C, and all these species reached maximum values at IN107 (*p*<0.05). Whereas PE32∶3, PE34∶6, PE36∶6 and PE36∶5, negatively correlated to the third component, reached their highest values in IN105.

Although PC was the most abundant class of phospholipids and the second most diverse lipid class in *C. sorokiniana*, it was less important than PC and PG in PLS-DA analysis (Figure 4C), with less inoculum-associated changes than PG and PE. Figure 8 showed the change of all the key PC species under different inoculum sizes. The most abundant PC contained the acyl chain moite of C34∶2. Thus, the phospholipids composed of C16 and C18 acyl chains might account for the majority of the most abundant phospholipids species in *C. sorokiniana* [28]. PC with short acyl chains, such as PC32∶3, PC34∶6, PC34∶5 and PC34∶2, were relatively stable in exponential phase while their contents were decreased with increasing inoculum sizes in the stationary phase. In contrast, the concentration of the long chain PC in IN106 was extremely higher than the others (*p*<0.05).

As aforementioned, the effect of inoculum sizes on phospholipid profiling of *C. sorokiniana* was mainly observed in IN106, with increased PG and PC, and reduced PE. Lots of evidence suggested that content distribution of phospholipids could change with the variation of temperature, light condition and nutrition supplement [61], [62], [63]. PC is the main component in plasmalemma system such as mitochondria and endoplasmic reticula, and it is also the minor phopholipid in chloroplasts. Moreover, PC is necessary for form of the DAG backbone of the glycolipids and it could be hydrolyzed by phospholipase D (PLD) to produce PA, acting as an important second messenger in response to various biotic and abiotic stresses [54], [64].

PE is another major constituent of microalgal plasma membranes. Some PE species are induced in plant cultures under anoxia stress and increased level of PE may provide some additional protection [27], [65], [66]. Membranes with high PE content can undergo laminar-hexagonal transition, which could not only affect membrane-membrane contact and bilayer fusion during processes of vesicle formation and vesicle-mediated protein trafficking [67], but also involve the integration of proteins to membranes, lateral movement in the membrane and folding and stabilization of certain protein complexes [68]. The significant increase of PE in IN106 might indicate a good growth status of *C. sorokiniana*.

As the only structural phospholipid in thylakoids and inner envelope membrane, PG was found to be essential for the dimerization of photosystem II(PSII) [69] and trimerization of Light Harvesting Complexes II (LHCII) [70] and PSI [71]. Recent research of the crystal structure of PSII from a cyanobacterium *Thermosynechococcus elongatus* revealed that PG served as additional lubrication for removal and insertion of CP43, a component of PS II in higher plants and microalgae. Furthermore, two PG species were located at the side of plastoquinone-plastoquinol (PQ-PQH2) exchange cavity and covered by a loop of D2, indicating PG might play an important role in oxygen diffusion to the cytoplasmic side of PSII [72]. Yu and Benning constructed a sulfoquinovosyl diacylglycerol (SQDG) and PG-deficient double mutant resulting in pale yellow cotyledons and leaves with reduced chlorophyll content and a severely compromised growth with an impaired photosynthetic capacity [73]. Pineau constructed two mutants of *Chlamydomonas reinhardtii* characterized by a remarkable reduction in their PG contents together with a complete loss in its Δ3-trans hexadecenoic acid-containing form, also lost PSII activity [74]. All these results indicated that PG not only was the essential component for formation of the thylakoid membranes, but also might be an important part involved in photosynthesis. Consequently, the change of phospholipids in this study might be in relation to inoculum-associated variation of the light condition in *C. sorokiniana* cultures. Apparently, the higher the inoculum size, the lower average light intensity would be for individual cells. However, the content of each PG species in IN107 was much lower than the one in IN106, because the change of light condition was not the only culture environment factor affected by inoculum sizes. Thus, PG could be potential metabolic engineering targets by engineering the key genes in the PG biosynthesis and metabolism pathways.

Tian has observed the phenomenon of the nutrition deficiency and cell stress caused by high inoculum size in yeast cells [27]. We have already analyzed the differences in the metabolite fingerprints against the metabolite variations of inoculum sizes, compared with those of *C. sorokiniana* in IN106 [28]. It’s clear that almost all detected metabolites in IN106 relatively had higher abundance than those under other conditions, especially in exponential phase. We also found that the highest photosynthesis-related protein expressions were mostly detected under the inoculum sizes of 1×10^6^ cells mL^−1^, which suggested the culture under this inoculum size exploited more protein synthesis potency of *C. sorokiniana* [31]. Combing all these results, the growth condition in IN106 was probably better than the other culture conditions in this study. Our results also suggested that culture process optimization of *C. sorokiniana* could be carried out based on the inoculum sizes of IN106; based on our previous study, the production per day of microalgae biofuel from *C. sorokiniana* was increased with increasing inoculums sizes [28]. Considering the changes of biofuel production [28] and photosynthesis related proteins [31] and phospholipids in this study according to inoculums sizes, it could be proposed that although activities of metabolism and photosynthesis were weaker than in IN106, the maximum biofuel productivity was observed in IN107 due to faster growth. Thus, the light limitation and cell stress displayed in IN107 suggested that careful selection of a reasonable inoculum size might be an important approach to improve the growth and biofuel production of *C. sorokiniana*.

### Conclusions

Research has shown high accumulation of phospholipids as a percentage of their total lipids mass in photosynthetic microalgae and suggested that lipids (biofuels) are the primary target for processors engaged in microalgae cultivation [75]. Thus purpose of this work was to identify the phospholipid profiling in *C. sorokiniana*, and then to find the effect of inoculum sizes on phospholipid profile. The phospholipidome under different inoculum size cultures was investigated by LC-MS. PLS-DA analysis showed a clear discrimination from the culture with the inoculum sizes of 1×10^6^ cells mL^−1^ to the others; and the key phospholipids responsible for this separation were PG and PE, which means the degree of membrane density and the fluidity of thylakoid membrane might play an important role in inoculum-associated mircoalgal growth and metabolism, and thus thylakoid membrane would be potential metabolic engineering targets for improvement of biofuel production and productivity from industry microalgae.
